# Editorial to “Utilizing the lid of SL sheath packaging for a water seal catheter insertion technique”

**DOI:** 10.1002/joa3.70028

**Published:** 2025-02-24

**Authors:** Mitsuru Takami, Kimitake Imamura, Koji Fukuzawa

**Affiliations:** ^1^ Section of Arrhythmia, Division of Cardiovascular Medicine, Department of Internal Medicine Kobe University Graduate School of Medicine Kobe Japan

Radiofrequency ablation, cryoballoon, hot balloon, laser balloon ablation, and, more recently, pulsed field ablation have been developed to improve the efficacy, shorten the procedure time, and enhance the safety. However, all ablation devices follow the same process: they are inserted from outside the body, where air is present, into blood vessels and the heart, where no air exists. This always carries the risk of air bubble intrusion. Newer ablation devices, like balloon‐based and pulsed field ablation devices, require larger sheaths and complex catheter shapes, increasing the risk of air bubble intrusion. To minimize the risk of this iatrogenic complication, ablation procedures must be performed with the utmost care and attention.

In this article, Hayashi et al.^1^ reported a novel method to prevent air bubble intrusion. They focused on the packaging of the SL sheath (Swartz™ Braided Transseptal Guiding Introducers SL Series, Abbott, Minneapolis, MN, USA) and demonstrated that by cutting a portion of the lid and filling it with water, an air seal can be created when inserting the catheter into the sheath. They also presented a video demonstrating this method, showing that the catheter can be inserted while keeping the sheath's entry completely submerged in water. According to the image, a slight bend may be necessary to fully submerge the sheath insertion site. However, they reported that the sheath tip remained stable in the left atrium, and after over 500 cases without any complications, they consider the technique highly safe.

The size and number of air bubbles responsible for symptomatic or asymptomatic embolisms in humans remain uncertain. However, larger air bubbles can significantly impact the cerebral and systemic circulation. For instance, the mean diameter of the proximal segment of the cerebral posterior communicating artery is 1.4 ± 0.5 mm. Larger air bubbles could obstruct these vessels, potentially leading to a cerebral infarction. Previously, we conducted an ex vivo study to identify the stages of catheter ablation most prone to air intrusion.^2^ Our findings indicated that massive and large (≥1.5 mm) air intrusion was most likely to occur when inserting a complex‐shaped catheter into the sheath under negative pressure in the left atrium (LA) using an inserter. In humans, the LA pressure is usually positive; however, studies have shown that negative pressure can develop in the LA (Inspiratory mean LA pressure: −3.1 ± 9.3 mmHg) during snoring caused by sedation.^3^ At that moment, catheter insertion into the sheath poses the highest risk of a massive air intrusion. Hayashi et al.'s method provides a simple technique with the potential to reduce air intrusion at this critical moment.

Another notable aspect of their method is the use of the plastic tray from the SL sheath packaging, which is usually discarded. Their strong desire to improve the safety of catheter ablation led them to solve the problem using only what is available in the cath lab. Moreover, their idea requires no extra cost and can be implemented immediately in any hospital, even amid current financial challenges in healthcare.

We would like to once again express our respect for Hayashi et al.'s insights and ideas. Since this report is not a comparative trial, a future comparative validation could further enhance its value.

## CONFLICT OF INTEREST STATEMENT

The Section of Arrhythmia (Kobe University Graduate School of Medicine) is financially supported by an endowment from Abbott Japan, Boston Scientific Japan, and Medtronic Japan. K.I. and K.F. belong to the Section and receive a scholarship donation from Biotronik Japan. M.T. is conducting joint research with Murata Manufacturing Co., Ltd. and Boston Scientific Japan. However, all authors report no relationships relevant to the contents of this manuscript.
